# Flexible Sensor Film Based on Rod-Shaped SWCNT-Polypyrrole Nanocomposite for Acetone Gas Detection

**DOI:** 10.3390/polym15163416

**Published:** 2023-08-16

**Authors:** Hyo-Kyung Kang, Jun-Ho Byeon, Hyun-Jun Hwang, Yoon Hee Jang, Jin-Yeol Kim

**Affiliations:** 1 School of Advanced Materials Engineering, Kookmin University, Seoul 02707, Republic of Korea; rkdgyrud7274@kookmin.ac.kr (H.-K.K.); qus0414@naver.com (J.-H.B.); hhjun0604@kookmin.ac.kr (H.-J.H.); 2 Advanced Photovoltaics Research Center, Korea Institute of Science and Technology (KIST), Seoul 02792, Republic of Korea

**Keywords:** flexible gas sensor, SWCNT-PPy composite, acetone gas sensor, polyimide substrate, room temperature

## Abstract

A nanocomposite rod-shaped structure with a single-walled carbon nanotube (SWCNT) embedded in polypyrrole (PPy) doped with nonafluorobutanesulfonic acid (C4F), SWCNT/C4F-PPy, was synthesized using emulsion polymerization. The hybrid ink was then directly coated on a polyimide film interdigitated with the Cu/Ni/Au electrodes via a screen-printing technique to create a flexible film sensor. The sensor film showed a response of 1.72% at 25 °C/atmospheric pressure when acetone gas of 5 ppm was injected, which corresponds to almost 95% compared to the Si wafer-based array interdigitated with the Au electrode. Additionally, C4F was used as a hydrophobic dopant of PPy to improve the stability of humidity and to produce a highly sensitive film-type gas sensor that provides stable detection even in humid conditions.

## 1. Introduction

Gas detectors have been a subject of great interest over the past few decades in response to industrial and medical demands in air quality monitoring [1], the automotive [2] and semiconductor industries, and in the screening or monitoring of various diseases [3,4,5,6]. Basically, a gas sensor consists of a gas-activation layer and a substrate interdigitated with electrodes. Most gas sensor substrates consist of alumina plates [7], ceramic plates [8], or silicon wafers [9], and the gas-sensing layers consist of metal oxides [10,11,12], such as ZnO [10], In_2_O_3_ [11], SnO_2_ [12], and WO_3_ [13]. Recently, flexible gas sensors [14,15,16,17,18,19,20,21] based on plastic substrates, such as polyimide (PI) [12,13,21], polycarbonate (PC) [16], and polyethylene terephthalate (PET) [20,21,22,23] films have been developed to achieve lower manufacturing costs as well as improved miniaturization, portability, and wearability. In addition, gas sensors that use π-conjugated carbon compounds (CCC), such as carbon nanotube (CNT) [20,21,22,23], polypyrrole (PPy) [17,24,25,26], polyaniline (PANi) [18,27,28], and graphite [29], as a gas-sensing layer are being actively studied. These CCC-based sensors exhibit improved features for gas detection [15,18,19,20,21,22,23,24,25,26]. They operate at low temperatures and the sensing material can be designed to detect selected compounds with a very low response to other compounds. Furthermore, they can be processed into thin films using a screen-printing process. Kumar et al. [18] described an ammonia gas sensor based on PANi as a gas-sensing layer deposited on a PET film and investigated the ammonia gas-sensing properties of the film at 25 °C from 5 to 1000 ppm. Hua et al. [23] reported a potential gas sensor for NH_3_, NO, and NO_2_ sensing applications using a single-walled CNT–Fe_2_O_3_ (SWCNT-Fe_2_O_3_) composite film deposited on a PI film substrate. Additional research into the potential applications of flexible gas sensors is ongoing, and new manufacturing technologies, such as screen printing and flexible film-forming processes, are critical to produce cost-efficient sensors.

**Figure 1:**
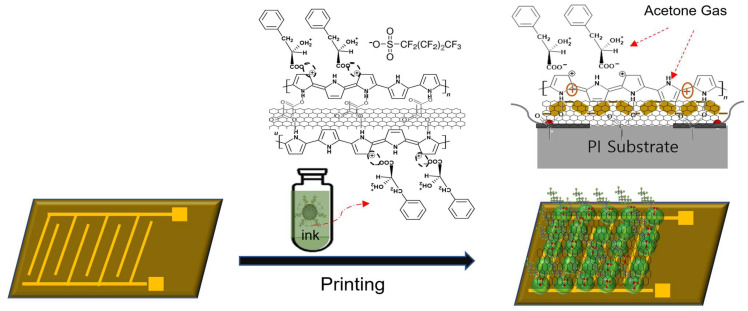
Top view and cross-section schematic diagram of a flexible sensor film with SWCNT/C4F-PPy nanocomposite layers on an IDE polyimide substrate (left: IDE substrate, right: flexible gas sensor film). The IDE substrate consists of two contact pads (2 mm × 2.5 mm) and a sensor electrode (width: 0.5 mm, distance: 0.8 mm) composed of the three Cu/Ni/Au layers. The sensor area is 8 mm × 10 mm. The sensor film on the right shows the layers composed of a rod-shaped SWCNT/C4F-PPy nanostructure.

In this work, we first fabricated an interdigitated electrode (IDE) with a Cu/Ni/Au three-layer structure on a flexible 50 µm thick PI film (IDE PI film). Second, a film-type flexible gas sensor was fabricated using a nonafluorobutanesulfonic acid-doped polypyrrole (C4F-PPy) and SWCNT nanocomposite ink, i.e., SWCNT/C4F-PPy, on an IDE PI film by screen printing to detect acetone gas. Figure 1 depicts the schematic diagram of a flexible gas sensor film with gas-sensing layers on an IDE PI substrate. Screen printing is advantageous due to its low cost, high-speed patterning, and applicability to various substrates, making it an efficient alternative to conversion patterning techniques used in the production of multi-purpose electronic devices. In particular, screen-printed sensor films can offer more benefits in terms of mass production, the control of sensing dimensions, and flexibility. The SWCNT/C4F-PPy nanocomposite sensor film was able to detect less than 1 ppm of acetone gas at 25 °C and exhibited moisture resistance. These SWCNT/C4F-PPy nanocomposite-based sensor films showed a response of 1.72% at 25 °C/atmospheric pressure when injected with 5 ppm acetone gas, which is nearly 95% compared to that of the Si wafer-based array interdigitated with Au electrodes.

## 2. Materials and Methods

### 2.1. Fabrication of PI Film Interdigitated with Cu/Ni/Au Electrodes: IDE Substrate

**Figure 2:**
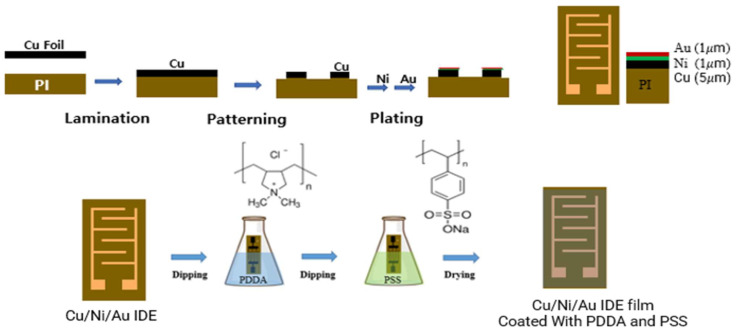
Schematic showing the preparation of the PI substrates interdigitated with the Cu/Ni/Au electrodes (IDE substrate). The IDE electrode lines have a distance and width of 0.8 mm and 0.5 mm, respectively. The PI substrate used has 50 µm thick film. The prepared IDE substrate film was treated with PDDA and PSS via a layer-by-layer technique to obtain a negatively charged surface layer, which improves the adhesion properties between the sensing material and the upper layer.

The fabrication process of the IDE substrate is shown in Figure 2. It was designed based on a 50 μm thick flexible PI film (Kapton, Dupont, Wilmington, DE, USA), where the electrode geometry is set to a level that enables higher sensor responsibility. First, the flexible PI film and 6 μm thick Cu foil, which is treated with hot melt adhesive, were bonded using a hot lamination process at 200 °C. Then, the Cu-laminated PI film was patterned via a photolithography process, and finally, 2–3 µm Ni and Au layers were sequentially formed on the Cu layer using electrolytic plating. The Cu-laminated PI film was immersed in an acidic solution (40 g/L of sulfamic acid) for 30 min to activate the surface. After that, it was immersed for about 30 min at a current density of 1 A/dm^2^ (PH 4) using the electrolytic plating method, and the Ni and Au layers were plated sequentially to a thickness of 1μm each. The result was an IDE substrate film consisting of two contact pads (2 mm × 2.5 mm) and a sensor electrode (width: 0.5 mm, distance: 0.8 mm) composed of a Cu/Ni/Au three-layered metal. Here, the Au in the surface layer of the three-layered electrode was configured to prevent oxidation and improve electrical conductivity, thereby providing an electrical resistance of 0.1 Ω·cm. Then, the as-prepared IDE substrate film was pre-treated with polycationic poly (dimethyl-diallyl ammonium chloride) (PDDA, Sigma-Aldrich, St. Louis, MI, USA) aqueous solution (1%) and a polyanionic poly (sodium 4-styrenesulfonate) (PSS, Sigma-Aldrich) aqueous solution (2 mg/mL; pH ≈ 1 adjustment) was sequentially applied using a layer-by-layer technique to obtain a negatively charged surface layer, as shown in Figure 2.

### 2.2. Synthesis of SWCNT/C4F-PPy Nanocomposite

**Scheme 1:**
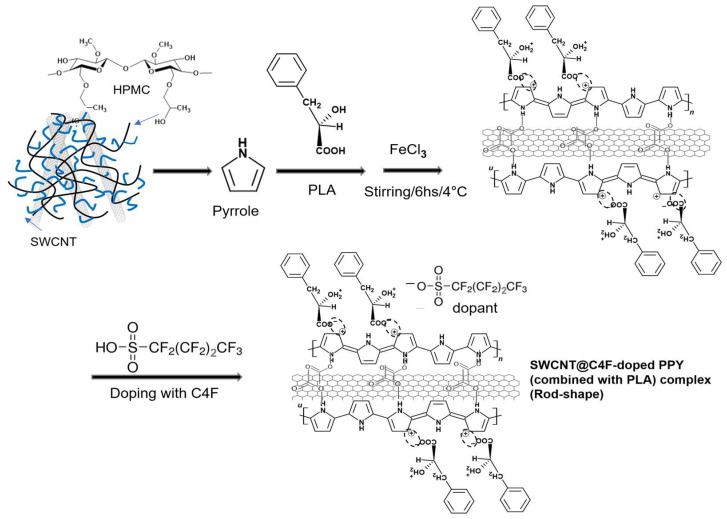
Synthetic procedure for the fabrication of a water-dispersible rod-shaped SWCNT/C4F-PPy nanostructure.

The rod-shaped SWCNT/C4F-PPy nanocomposite was synthesized using an emulsion polymerization technique described in a previous paper [26]. The synthesis proceeded in five steps. First, hydroxypropyl methylcellulose (HPMC, Sigma-Aldrich) was dissolved in deionized (DI) water to prepare 1 wt% dispersion solution. Conductive SWCNTs were purchased from Zeon (0.5 wt%, aqueous solution) and diluted without any treatment. Then, 0.0031 g of the conductive SWCNTs was added to 30 mL of HPMC solution and sonicated for 30 min. Second, 0.3 g of pyrrole monomer and 0.37 g of L-(-)-3-phenyllactic acid (PLA) were each dissolved in 30 mL of HPMC and SWCNT mixed solutions in order and stirred at 0 °C for 30 min. Third, 3.11 g of iron chloride hexahydrate (FeCl_3_·6H_2_O), as an oxidant, was added to the mixture, and reacted at 0 °C for 6 h to complete the synthesis of the final solution. Finally, 560 uL of C4F (40 wt% aqueous solution) (pyrrole: C4F = 1:0.1 molar ratio) was added as a dopant and the mixture was stirred for more than 12 h. Scheme 1 provides a representation of the synthesized rod-shaped SWCNT/C4F-PPy nanostructures. The synthesized nanostructure was purified 2 to 3 times using ultracentrifugation. The synthesized rod-shaped SWCNT/C4F-PPy nanocomposite (3 wt%) was prepared as an ink redispersed in a mixed solvent of DI water–isopropanol (1:1 *v*/*v*) with 2 wt% HPMC and 3 wt% PVP. An ink solution using the SWCNT/C4F-PPy nanocomposite was prepared to have a viscosity of 6000–8000 cps for screen printing. 

### 2.3. Performance Evaluation of Gas Sensors

The ink solutions using the as-synthesized SWCNT/C4F-PPy nanocomposite were coated on the prepared IDE substrate strip through screen printing technology to form a sensing layer, and then dried in a vacuum oven at 60 °C for 1 h. The prepared sensor strip (test cell) was installed in the 350 mL gas chamber. The chamber was purged with dry air, which was introduced into the chamber by a carrier gas, for 10 min while maintaining the chamber pressure at atmospheric pressure. Finally, acetone gas in varying concentrations (1–5 ppm) was introduced. The response (S) of the acetone gas was determined from the difference between the initial (*R*_0_) and current resistance (*R*). A sensor array film was used to detect acetone gas, where S was defined as Δ*R*/*R*_0_ × 100 (Δ*R* = *R* − *R*_0_). Then, the acetone gas was exposed to concentrations of 1 to 5 ppm at 25 °C, in dry air (0% relative humidity (RH)) and wet conditions (10 to 80% RH/25 °C). The resistances between the electrodes were measured under the constant DC applied current. The current resistance (Ω, ohm) was measured using a resister (Keithley 2000) by applying a DC voltage of 4 V. Gas-sensing characteristics were also observed under various humidity conditions from 0 to 85% RH and temperature conditions of 25 °C, 40 °C, and 55 °C, respectively.

## 3. Results and Discussion

The overall synthetic procedure used to create the water-dispersible SWCNT/C4F-PPy nanocomposite is described in Scheme 1. First, an aqueous HPMC solution where conductive SWCNTs were uniformly dispersed was prepared for emulsion polymerization, and then pyrrole monomer and PLA were sequentially added to it. FeCl_3_ was then added as an oxidizing agent to initiate the polymerization reaction. This resulted in the formation of a rod-shaped nanostructure with SWCNT at the core surrounded by PPy–PLA (Scheme 1). A previous study reported that PLA is chemically bonded with the PPy chain and reacts selectively with a specific gas such as acetone [26]. The resulting products can maintain long-term dispersion stability in aqueous solution without precipitation due to its excellent dispersion characteristics. Structurally, SWCNTs surface-treated with COOH are chemically interconnected with NH groups of the pyrrole ring and can also electrically transfer electrons. SWCNTs with high electrical conductivity can greatly increase electrical conductivity by extending the electron transport path with PPy, which is an electrically conductive polymer chain. Notably, conductive SWCNTs have been shown to improve the electrical conductivity of PPy by more than 30%. A resistance value of 24 KΩ·cm was measured on a 10 µm thick SWCNT/C4F-PPy film. For screen printing, the synthesized SWCNT/C4F-PPy nanocomposite (3 wt%) was redispersed in a mixed solvent of DI water–isopropanol (1:1 *v*/*v*), where 2 wt% HPMC was dissolved to prepare a coating ink.

**Figure 3:**
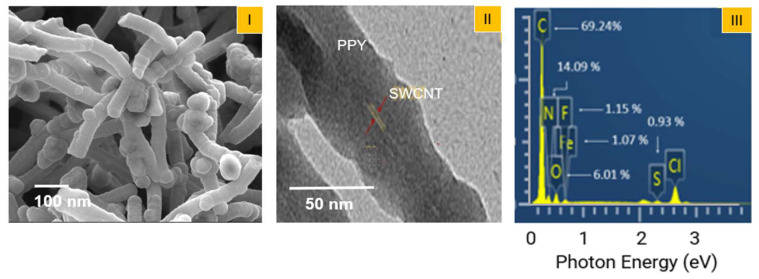
(**I**) SEM and (**II**) TEM morphologies of SWCNT/C4F-PPy core–shell-shaped nanorods. (**III**) EDS spectra showing the elemental analysis data for SWCNT/C4F-PPy. Red arrow in (**II**) indicates the SWCNT core.

Figure 3I,II show the morphological and structural characteristics of the SWCNT/C4F-PPy nanostructures printed on the IDE PI film investigated by SEM and TEM. The TEM of Figure 3II is an enlarged image selected from the area of the specified part of the SEM of Figure 3I. As shown in figure, their nanostructures exhibited well-defined rod-like morphologies with a length of about 3 μm and an average diameter of 60 nm. In particular, the SWCNT/C4F-PPy nanostructures have a core–shell structure surrounded by a C4F-doped PPy layer with a thickness of approximately 30 nm on the outer surface of the SWCNT single strand. The outer surface of the 2 nm thick SWCNT is covered by a layer of C4F-PPy with a thickness of about 15 nm, and a thin organic PLA layer of about 2.5 nm covers the surface layer of PPy. C4F, used as a hydrophobic dopant, was produced in the shell of the PPy rod during polymerization. Here is a C4F molecule containing fluorine (F) atoms that sufficiently covers the PPy’s surface. Energy-dispersive X-ray spectroscopy (EDS) was employed to examine the component analysis of the SWCNT/C4F-PPy, as demonstrated in Figure 3III. The analysis showed that the contents of major atoms such as C, O, N, S, and F were 62.24%, 6.01%, 14.09%, 0.93%, and 1.15%, respectively. The mass percentage of F atoms on the SWCNT/C4F-PPy was estimated and compared with the content of other components. The mass percentage of F and S atoms was found to be 1.15% and 0.93%, respectively, for SWCNT/C4F-PPy. However, EDS data confirmed the presence of S and F atoms in the C4F molecule, which was used as the dopant.

**Figure 4:**
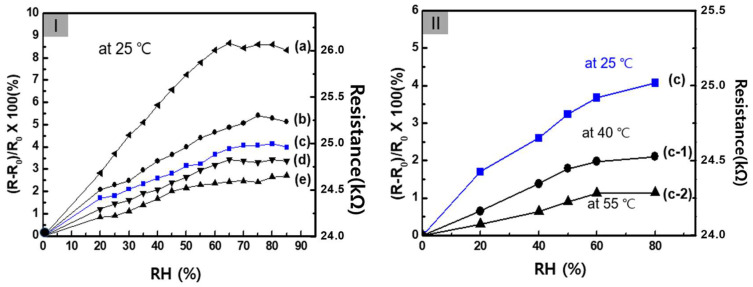
(**I**) Variation in sensor resistance (response) of SWCNT/C4F-PPy film exposed to different humidity conditions at 25 °C; the mole ratio of C4F is (a) 0, (b) 0.05, (c) 0.1, (d) 0.2, and (e) 0.3, respectively. (**II**) Electrical resistance of SWCNT/C4F-PPy sensor film with C4F content of 0.1 molar ratio (shown in blue line) with varying humidity at multiple temperature conditions: (c) 25 °C, (c-1) 40 °C, (c-2) 55 °C, respectively. The initial resistance (*R*_0_) of the samples used in the experiment is 24 KΩ·cm.

External humidity plays an important role in the stability and performance of biosensors. Therefore, obtaining a reliable response to humidity in biosensors and avoiding cross-response due to moisture remains a major challenge [30]. As is widely known, most organic-based sensors including metal oxide sensors exhibit lower response under humid conditions. Herein, we synthesized a hydrophobic PPy nanocomposite, SWCNT/C4F-PPy nanocomposite, using C4F as a dopant to ensure the stability of the sensor material in humid conditions. Previous researchers [31] studied the stability of a PPy-based acetone gas sensor in humid conditions using C8F as a dopant, and based on their results, we believed that C4F would also make the PPy surface hydrophobic and neutralize it, implying that C4F can be used to impart materials with water-repellent properties. Figure 4 shows the change in the electrical resistance of the SWCNT/C4F-PPy films as a function of relative humidity (RH). As shown in Figure 4I(a), the control of SWCNT/PPy film (no C4F) exhibited a significant change in resistance with an increase in the RH% at 25 °C. In contrast, resistance was rapidly stabilized as the C4F content increased to 0.05, 0.1, 0.2, and 0.3 molar ratios based on the pyrrole monomer (Figure 4I(b–e)). The variation in the sensor resistance under various humidity conditions also improved when the C4F content was 0.1 mol/ratio or more; however, the electrical resistance of the sensor film rapidly decreased. Since hydrophobic hydrocarbons containing a large amount of fluorine are attached to the PPy polymer chain, which is a conductive polymer, it is thought that if present in an appropriate amount or more, it becomes an obstacle and lowers the electrical resistance or sensitivity. However, on the contrary, it was observed that the stability of electrical resistance was greatly improved under high humidity conditions. Figure 4II shows the variation of sensor resistance of the SWCNT/C4F-PPy (C4F content: 0.1 molar ratio, shown in blue line in Figure 4I(c)) film at three different temperatures. Sensor resistance was measured in the range of 25−55 °C. As shown in the figure, the higher the measurement temperature, the more stable the change in sensor resistance. This improves the activation of carrier ions under high-temperature conditions, and as a result, a more stable gas-sensing performance with less resistance change according to high humidity conditions can be obtained.

**Figure 5:**
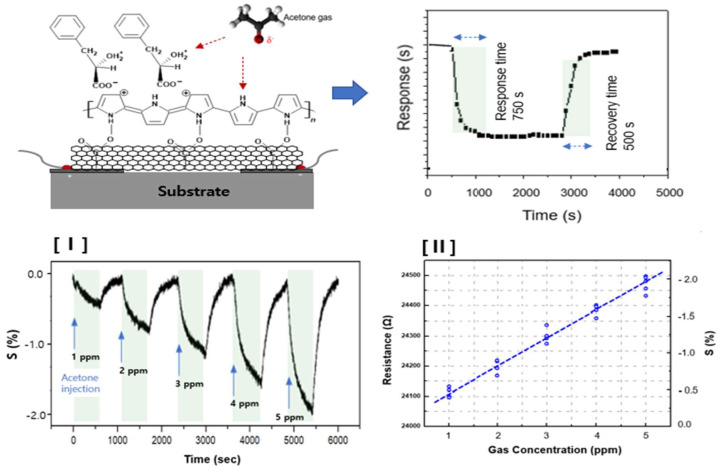
Schematic diagram of sensor array using SWCNT/C4F-PPy nanocomposite and dynamic response in sensors to various concentrations of acetone gas at 25 °C and 0% RH. It was observed that the sensor resistance increased when the sensor array film was exposed to acetone; the response time was 750 s and the recovery time was 500 s. (**I**) Response spectrum of the SWCNT/C4F-PPy nanocomposite sensor to different concentrations of acetone gas as a function of time. (**II**) Change in response sensitivity according to the concentration of acetone gas from 1 to 5 ppm. The sensitivity (ppm^−1^) of the acetone gas sensor can be calculated as 0.37.

We fabricated the gas sensor array by screen-printing a SWCNT/C4F-PPy nanocomposite ink solution onto the IDE substrate film (test cell: with a pair of integrated Cu/Ni/Au electrodes on a 50 µm thick PI substrate), as shown in Figure 5. Cu/Ni is a layer composed to give adhesion between Au and PI, and here, Ni is composed for the purpose of preventing the migration and oxidation of Cu. The Au layer is an electrode material that determines the actual sensor performance. The thickness was designed under the optimal conditions required to maintain the stable redox characteristics of the electrode. When the thickness of Cu/Ni/Cu is greater than necessary, it is difficult to secure the flexibility or bending stability of the substrate film, so thinner layers are required. In the case of PI-based IDE substrate film, the mechanical strength and high temperature stability are weaker than existing rigid substrates (alumina plates, ceramic plates, or silicon wafers), but large-area film processing is possible by roll coating or through the screen print process, and it has the advantage of being flexible and able to manufacture thin film sensors at low cost. Therefore, in the case of sensor-active materials composed of metal electrodes and metal oxide that require a sintering temperature of 400 °C or more as a subsequent process of the sensor substrate, it is difficult to apply the flexible IDE PI substrate. The surface morphology of the sensor layer made of one-dimensional SWCNT/C4F-PPy nanocomposite has a microporous structure in which a three-dimensional network appears to be entangled. It is known that PPy or SWCNT/PPy composite compounds show changes in electrical resistance according to the typical characteristics of p-type semiconductors [32,33]. In particular, positively charged carriers (so-called polarons) are distributed in the PPy polymer chain connected to the SWCNT surface, and when acetone molecules that act as acids come into contact with them, they can react chemically and change electrical resistance. Hence, the PPy interacts with acetone to gain an electron; this electron transfer between positively charged PPy and acetone causes an elevation in the charge-carrier concentration, which causes a decrease in the overall electrical resistance. In particular, the PLA molecule can more easily receive electrons from acetone, from which some of the hydroxyl groups are converted to OH_2_^+^, thereby increasing the positive charge density of the PPy backbone by self-doping. That is, PPy combined with PLA has a function of selectively detecting acetone gas. SWCNTs, which have high electrical conductivity, chemically bonded to PPy, providing a path for higher electron movement and greatly improving the electrical conductivity of the sensor film. However, the advanced electrical properties of the conductors were improved by maintaining good contact between the SWCNTs and PPy. In particular, one-dimensional conductive SWCNTs are used to fabricate nanocomposites with PPy due to the advantages of a larger surface area and better electronic properties. As a result, more effective gas-sensing performance could be obtained by combining SWCNT with PPy.

The sensor array film was placed in a gas chamber and the chamber was purged with pure dry air using a carrier gas for 10 min while maintaining the chamber at atmospheric pressure. Then, various concentrations of acetone gas were injected into the chamber under different temperature and humidity conditions. Electrical resistance (Ω/cm) was measured in real time using a digital multimeter to record changes corresponding to different gas inflow. The sensor array films were used to detect acetone vapor and normalized the electrical resistance change to evaluate the response. When the sensor array film was exposed to pure acetone gas, the electrical resistance increased rapidly and the response time was 750 s. Figure 5I shows the response characteristics of the sensor array to the injection of acetone gas in the range of 1 to 5 ppm. As shown in the figure, it was observed that the sensor resistance increased when the sensor array film was exposed to acetone. However, as shown in Figure 5II, it was observed that S had a linear characteristic toward an increase in resistance as the concentration of injected gas increased. At this time, the sensitivity (ppm^−1^) of the acetone gas sensor can be calculated as 0.37.

**Figure 6:**
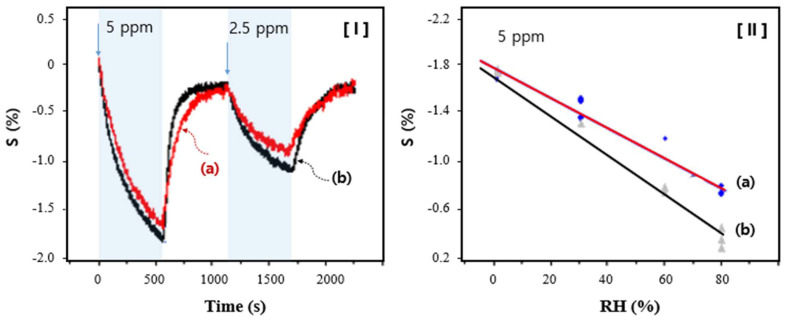
(**I**) Continuous dynamic response of the SWCNT/C4F-PPy nanocomposite sensor at different concentrations of acetone (2.5 and 5 ppm) at 25 °C/0% RH conditions. (**II**) Response changes with increasing humidity levels measured at 5 ppm acetone concentration under 25 °C. (a) PI film-based sensor integrated with Cu/Ni/Au electrodes and (b) Si wafer-based sensor integrated with Au electrodes.

Figure 6I(a) shows representative data to illustrate the response characteristics of the sensor array film to acetone gas concentrations of 2.5 and 5 ppm at 25 °C and 0% RH. Acetone concentrations of 2.5 and 5 ppm resulted in response (S) measurements of 0.92 and 1.67, respectively, and the relative response time was slightly faster at the higher concentration. At 5 ppm of acetone vapor, the S value of the sensor reached 1.67 within a response time of 750 s. For comparison purposes, Figure 6I(b) shows the acetone response data of a sensor fabricated by drop-casting SWCNT/C4F-PPy nanocomposite ink solution onto Si substrate with an integrated Au electrode. Although the Cu/Ni/Au-based PI substrate sensor (Figure 6I(a)) demonstrates approximately a 5% difference in response from the Au-based Si wafer substrate sensor (Figure 6I(b)), the results show a similar level of response characteristics. Figure 6II(a) plots the measured change in sensor response under various humidity conditions when 5 ppm of acetone gas was injected to the chamber. As shown in Figure 6II, S showed a tendency to decrease rapidly when the RH was 10% or more, and the change was generally linear. When the RH was at the highest level of 80%, S decreased to 0.72. Compared to the Si wafer-based sensor (Figure 6II(b), the PI-based film sensor (Figure 6II(a)) showed better response in the range of 10%–80% RH. Acetone gas was only weakly detected when RH was 80% or higher using the Si wafer-based sensor. These results indicate that the PI-based film sensor is better at sensing acetone gas in high humidity conditions.

Selectivity is another crucial parameter of gas sensors used in practice. To observe selectivity, we further tested various organic gases such as ammonia, toluene, ethanol, xylene, chloroform, hydrogen, and carbon dioxide. As a result, in the case of ammonia, on the contrary, the response (S) was observed as high as positive 2.0 (at 5 ppm) in the direction of decreasing electrical conductivity, but other gases measured relatively very low values of responsiveness less than 0.5. However, acetone gas showed negative responsiveness due to an increase in electrical resistance in molecules with high polarity. We noted that only acetone gas exhibited remarkable sensing characteristics in the direction of increasing electrical conductivity, unlike other types of gases. In addition, when gaseous molecules of Lewis acids such as acetone react to the positively charged PPy chain, the electrical resistance increases and the responsiveness is negative in direction. Conversely, it was observed that the gas molecules of Lewis bases such as ammonia decreased in electrical resistance and responded in a positive direction.

## 4. Conclusions

We synthesized a rod-shaped nanocomposite with SWCNT inserted into C4F-PPy emulsion polymerization and used it as an acetone-sensing material. SEM and TEM were used to analyze the structure of the nanorod-shaped SWCNT/C4F-PPy composite, and the contents of the constituent elements were confirmed from the EDS spectrum. A hybrid ink composition composed of SWCNT and C4F-PPy was directly coated onto a flexible polyimide film interdigitated with the Cu/Ni/Au electrodes using a screen print process. This sensor film provided a response of 1.72% in 5 ppm of acetone, closely mirroring the sensor response characteristics of the Si wafer substrate. However, the sensor film performed better in high humidity conditions. In this study, the flexible-type sensor film synthesized in the low-temperature/continuous film process demonstrated the potential for application across various fields.

## Data Availability

Not applicable.
